# Supplementary figures and images for: Establishment and maintenance of NRT2.1 inter-individual variability in plants
Source: PLoS Genet. 2025 Dec 17;21(12):e1011984. doi: 10.1371/journal.pgen.1011984 (PMC12742734; doi:10.1371/journal.pgen.1011984)

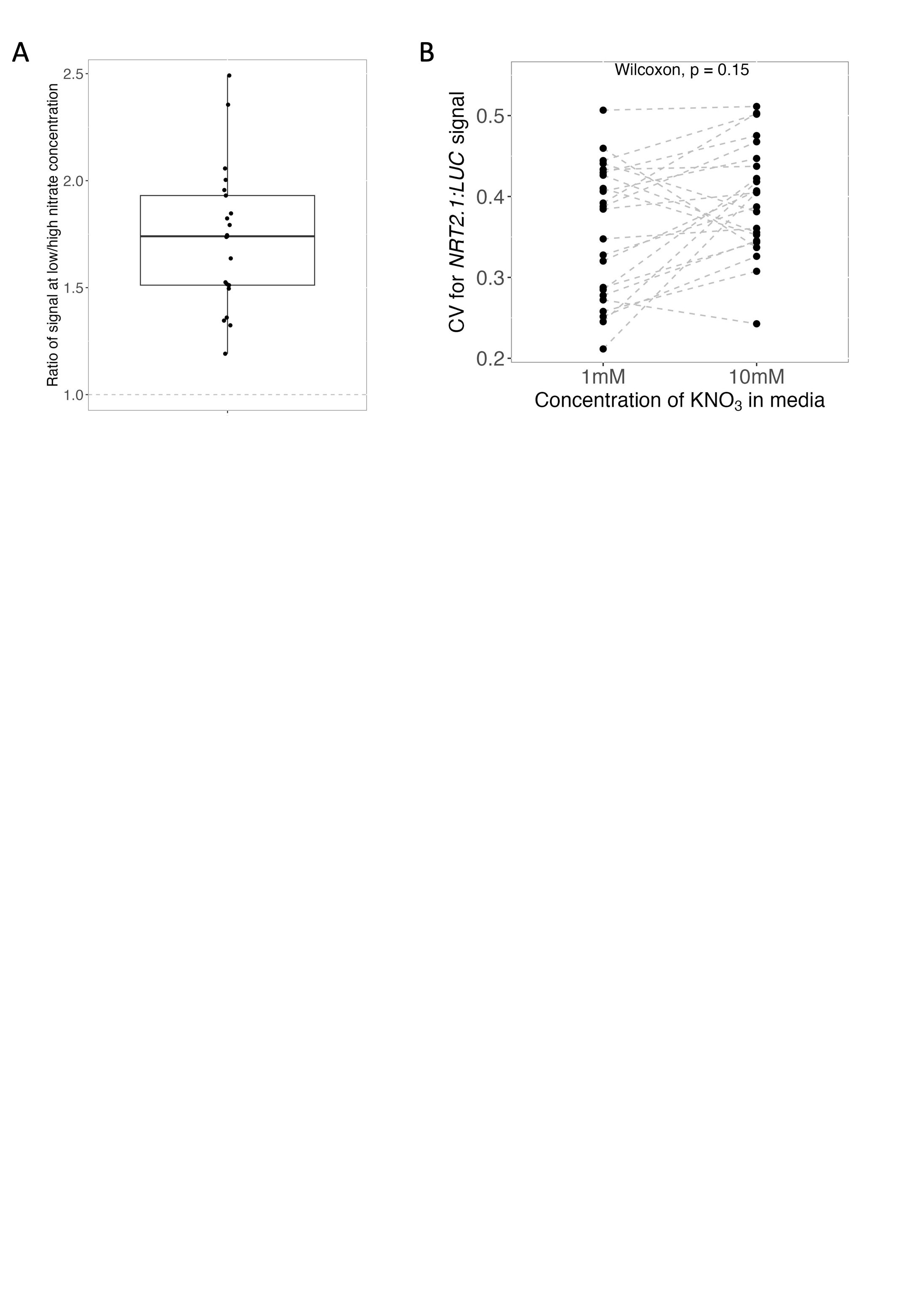

Supplement: S1 Fig — (A) Boxplot of the ratio of the pNRT2.1:LUC signal at low (1 mM) and high (10 mM) nitrate concentrations. Each point corresponds to an independent assay. The dotted line at 1 indicates an absence of difference in signal between low and high nitrate, which is not observed in any of the assays. (B) Inter-individual transcriptional variability of the pNRT2.1:LUC reporter line measured for seedlings grown on media with low (1 mM) of high (10 mM) nitrate concentration. Each point represents an independent assay with around 25 seedlings on low nitrate and 25 seedlings on high nitrate per assay. Points from the same assay are linked with a grey dotted line. (TIFF) [file pgen.1011984.s001.tiff]

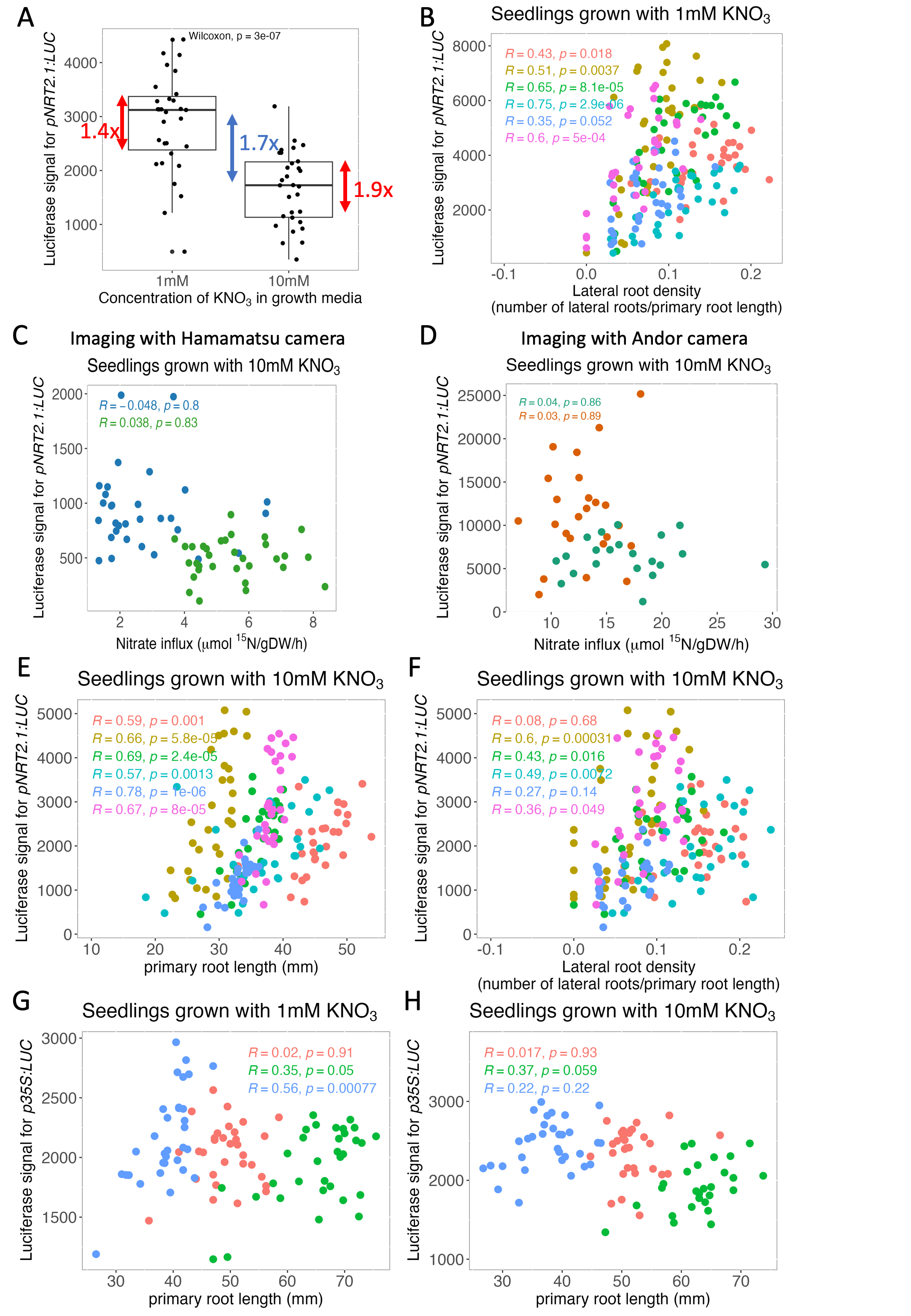

Supplement: S2 Fig — (A) Comparison for the pNRT2.1:LUC line of the differences in signal between seedlings in one condition (1.4x to 1.9x in red) and between the mean signal of seedlings grown in media with low (1 mM) or high (10 mM) nitrate concentration (1.7x in blue). Each point corresponds to the signal in a single seedling. The result of a Wilcoxon test comparing pNRT2.1:LUC signal at low (1 mM) or high (10 mM) nitrate concentration is also included. (B) Comparison for plants grown in media with low (1 mM) nitrate concentration of the pNRT2.1:LUC signal in the primary root and the lateral root density (number of lateral roots/primary root length). Each point corresponds to a single seedling and each colour to an independent assay. The result of a Spearman correlation test for each assay is included. (C-D) Comparison for plants grown in media with high (10 mM) nitrate concentration of the pNRT2.1:LUC signal and the nitrate influx of HATS. Each point corresponds to a single seedling and each colour to an independent assay. The Spearman correlation test result for each assay is included. Luciferase imaging was performed with (C) a Hamamatsu C4880-30-24W CCD camera for half of the assays and (D) an Andor iXon ultra 897 EMCCD Back-illuminated camera for the other half. (E-F) Comparison for plants grown in media with high (10 mM) nitrate concentration of the pNRT2.1:LUC signal at the primary root and (E) the primary root length or (F) the lateral root density (number of lateral roots/primary root length). Each point corresponds to a single seedling and each colour to an independent assay. The result of a Spearman correlation test for each assay is included. (G-H) Comparison for plants grown in media with (G) low (1 mM) or (H) high (10 mM) nitrate concentration of the p35S:LUC signal at the primary root and the primary root length. Each point corresponds to a single seedling and each colour to an independent assay. The result of a Spearman correlation test for each assay is included. (TI [file pgen.1011984.s002.tiff]

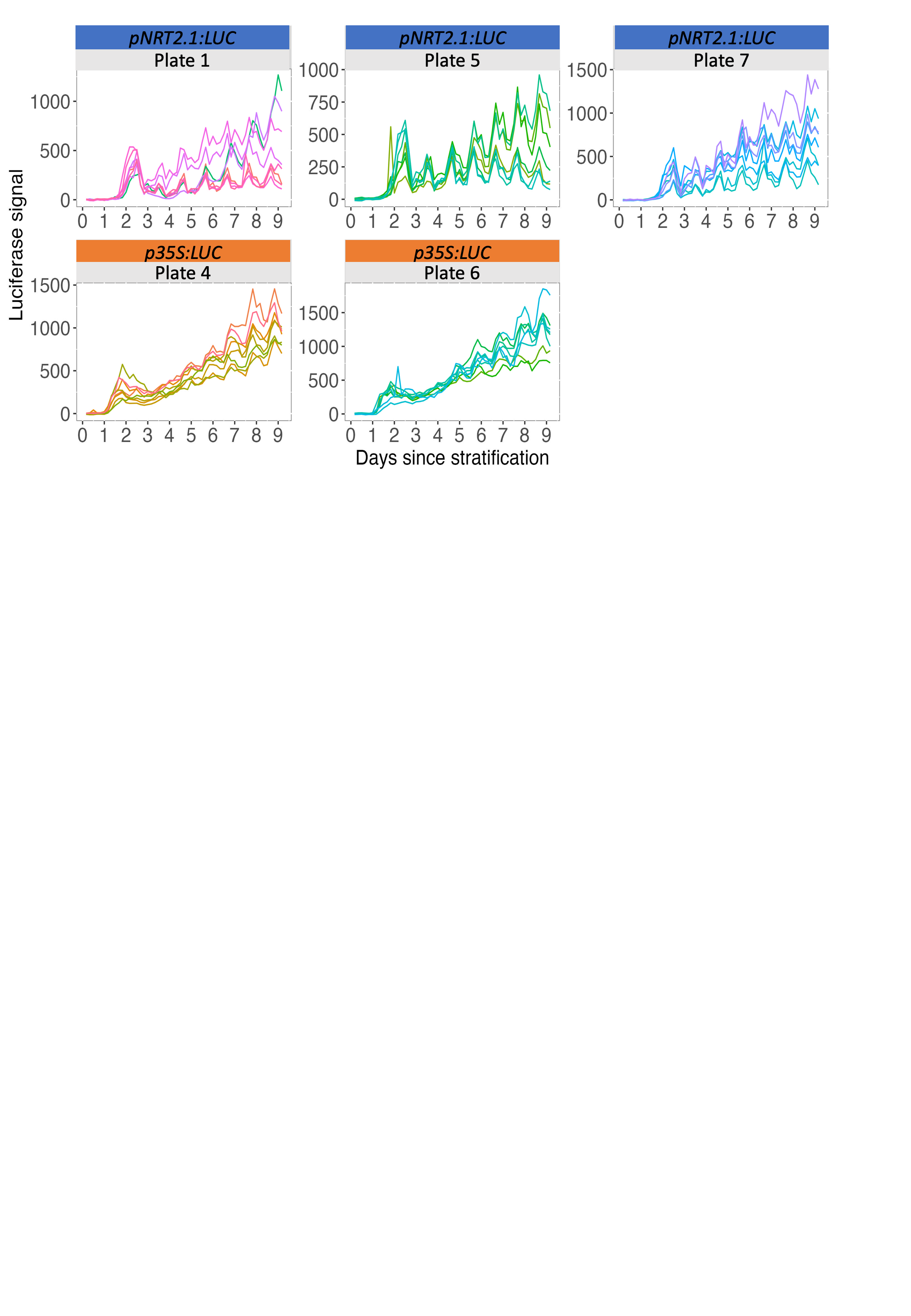

Supplement: S3 Fig — Seven to eight seedlings were measured in each plate. Only seedlings that have lateral roots at the end of the experiment were measured. Each line represents the signal in a given seedling, measured every 4 hours for 9 days after stratification. (TIFF) [file pgen.1011984.s003.tiff]

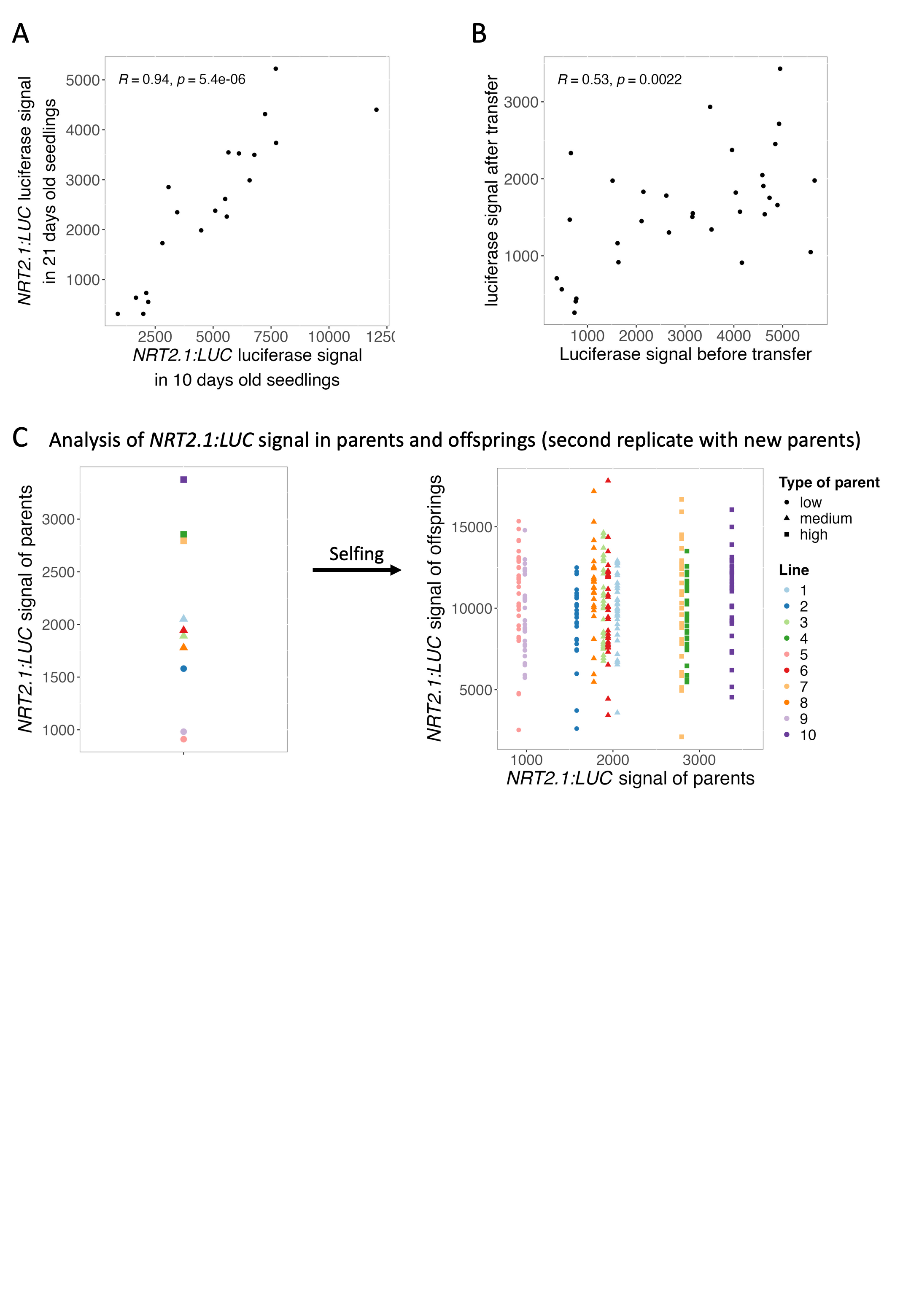

Supplement: S4 Fig — (A) Correlation of the pNRT2.1:LUC signal in 21-day-old and 10-day-old seedlings, corresponding to the data shown in Fig 4A. Each point corresponds to a seedling. The result of a Spearman correlation test is included. (B) Correlation of the pNRT2.1:LUC signal in seedlings before and after a transfer to a new plate with the same concentration of nitrate, corresponding to the data shown in Fig 4B. Each point corresponds to a seedling. The result of a Spearman correlation test is included. (C) Relation in pNRT2.1:LUC signal between parents and offsprings. Left: pNRT2.1:LUC signal for 10 seedlings (independent assay of Fig 4C). The individuals selected to analyse the signal of their offsprings are shown in shapes depending on their category: low expression as circles, medium expression as triangles, and high expression as squares. Right: distribution of the pNRT2.1:LUC signal for populations deriving from self-pollination of the parents selected. Each point corresponds to an individual and the different colours to a population of descendants, with the shape depending on the category of the parent: low expression as circles, medium expression as triangles, and high expression as squares. (TIFF) [file pgen.1011984.s004.tiff]

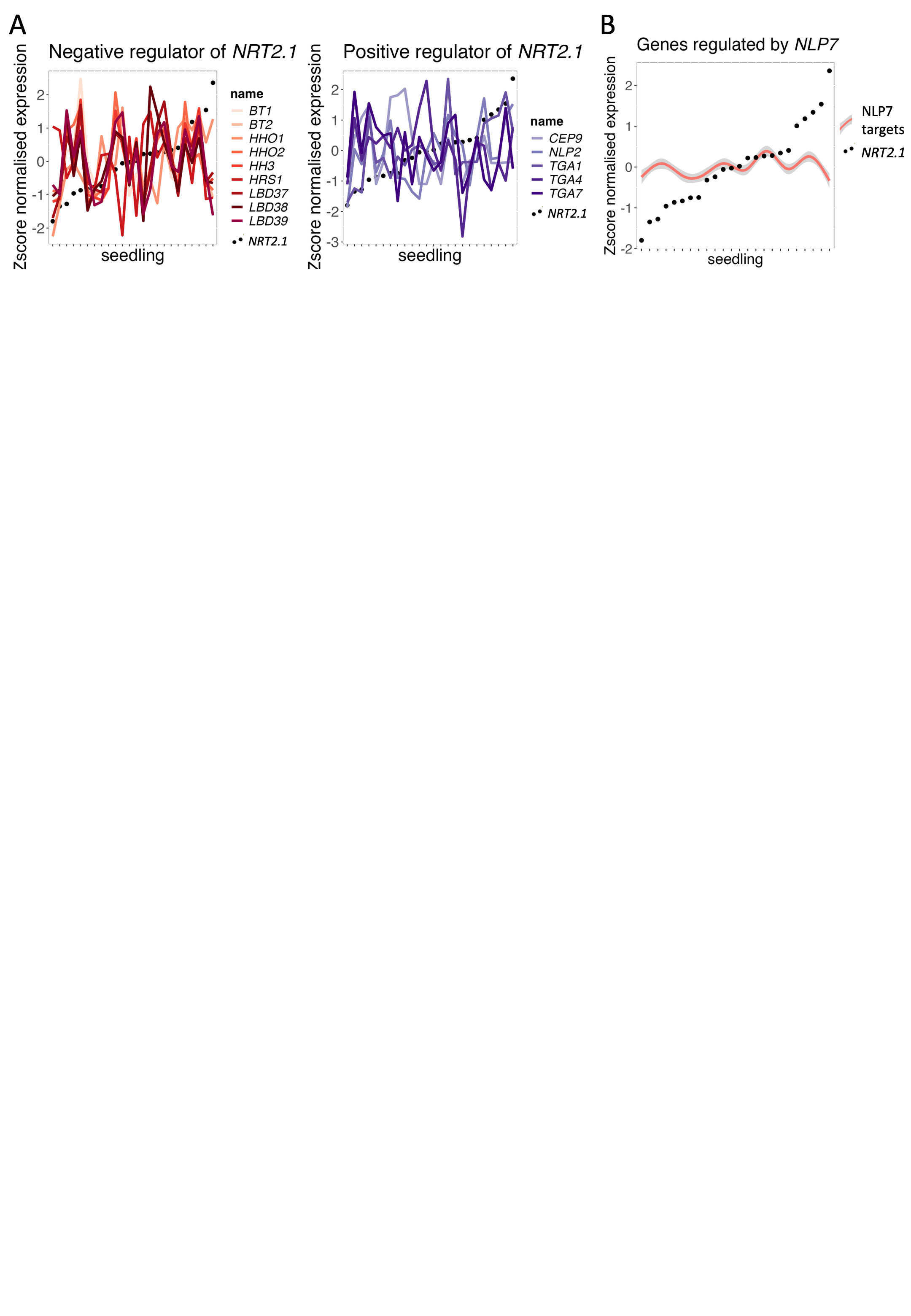

Supplement: S5 Fig — (A) Expression level in the different seedlings of NRT2.1 (black dots), and of negative regulators of NRT2.1 expression (shades of red, left), or positive regulators of NRT2.1 expression (shades of purple, right). Genes with a statistically significant correlation are in bold. (B) Expression in the different seedlings of NRT2.1 (black points), and of the average and standard deviation for genes targeted by NLP7 transcription factor. (TIFF) [file pgen.1011984.s005.tiff]

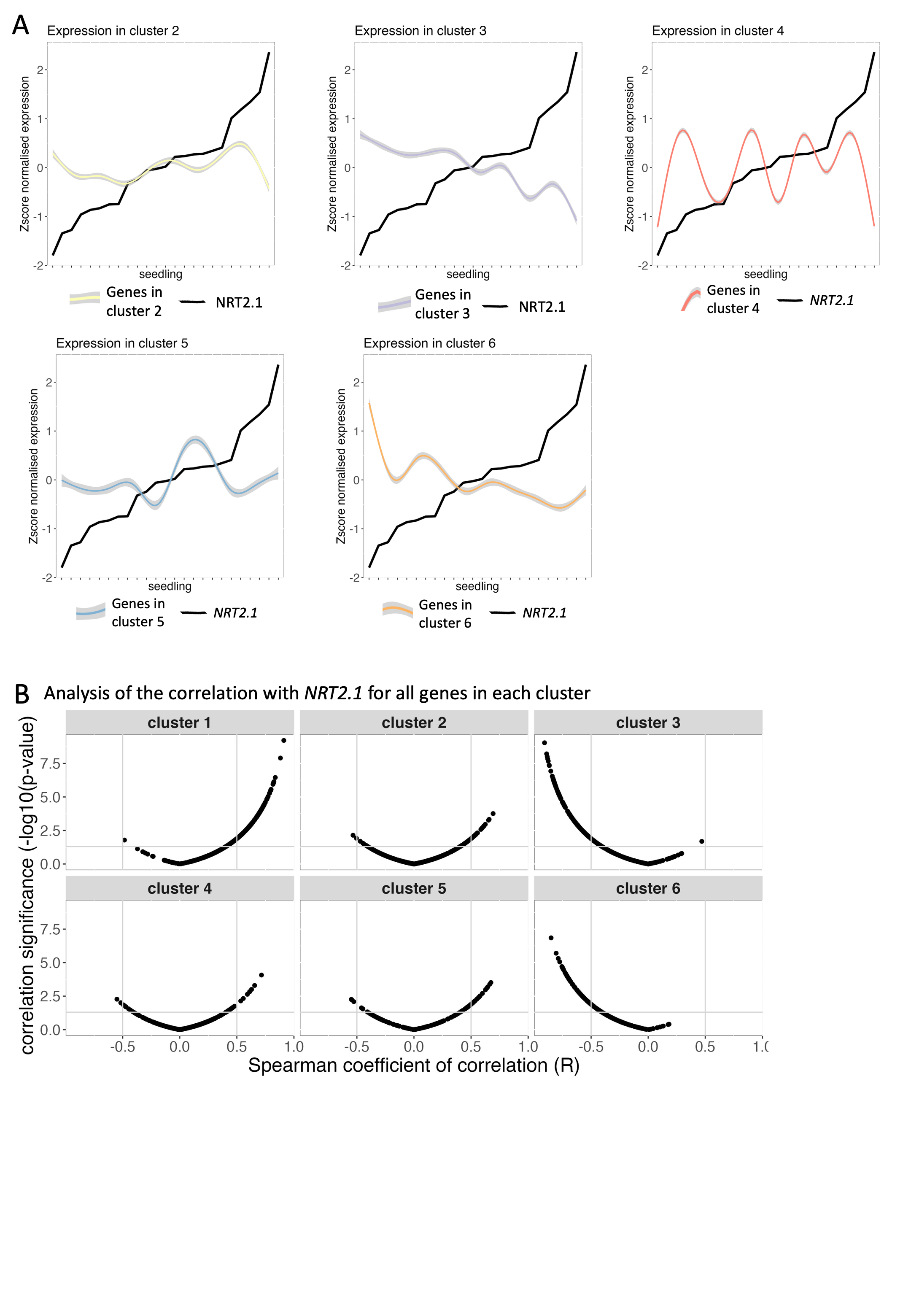

Supplement: S6 Fig — (A) Expression in the different seedlings of NRT2.1 (black lines), and of the average and standard deviation for the genes in each cluster identified in the hierarchical clustering shown in Fig 6A, with one plot per cluster. (B) Volcano plots, comparing the correlation significance (-log10(p-value)) with the Spearman coefficient of correlation (R) for the correlation with NRT2.1 expression in single seedlings, with one plot per cluster identified in the hierarchical clustering shown in Fig 6A. (TIFF) [file pgen.1011984.s006.tiff]

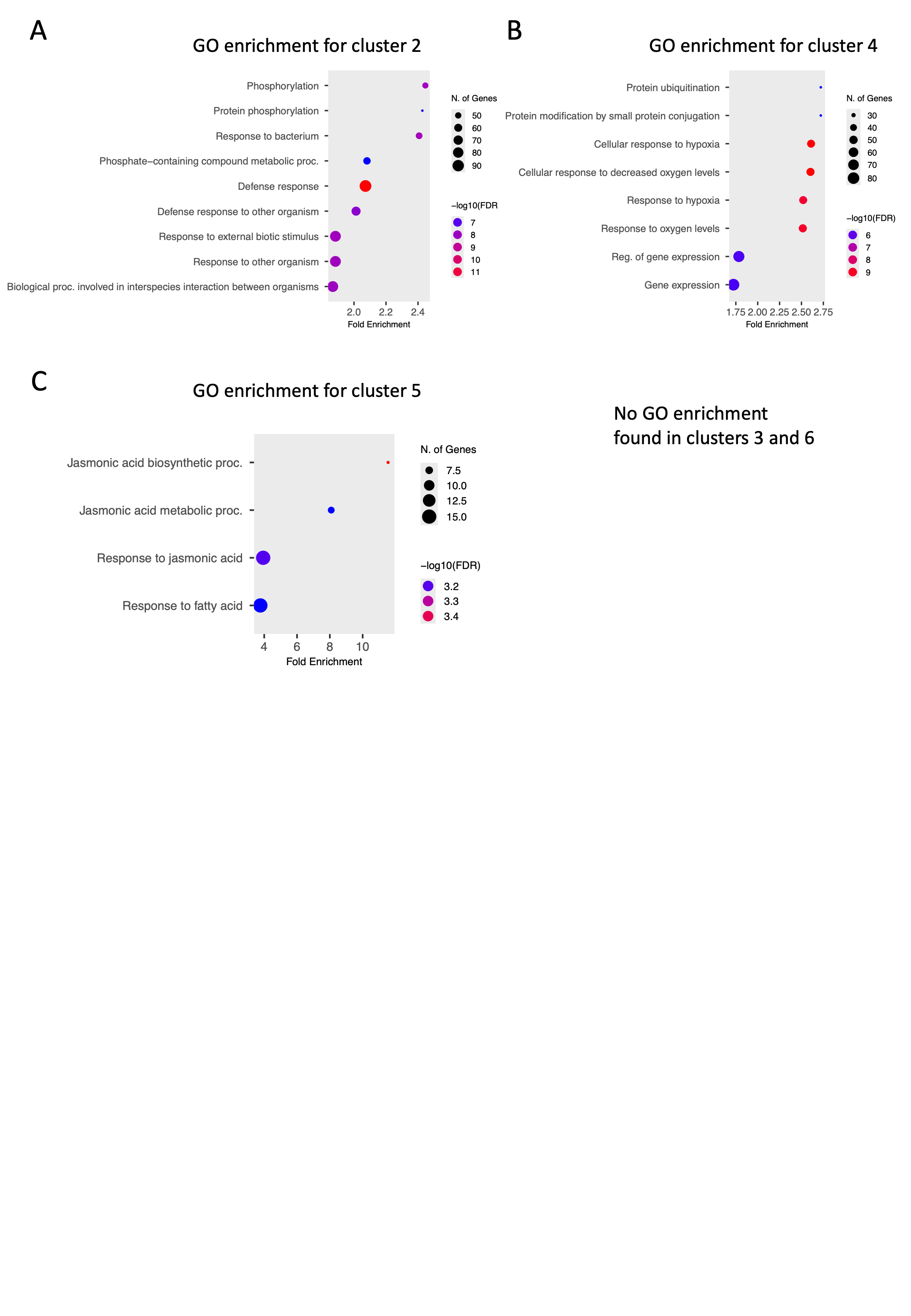

Supplement: S7 Fig — (A-C) GO enrichment analysis using genes in (A) cluster 2, (B) cluster 4 and (C) cluster 5 identified in the hierarchical clustering shown in Fig 6A. No GO terms were found to be enriched in other clusters (except cluster 1 shown in Fig 6B). (TIFF) [file pgen.1011984.s007.tiff]
