# Supplementary material for: Establishment and maintenance of NRT2.1 inter-individual variability in plants
Source: PLoS Genet. 2025 Dec 17;21(12):e1011984. doi: 10.1371/journal.pgen.1011984 (PMC12742734; doi:10.1371/journal.pgen.1011984)
Supplement: Attachment — Submitted filename: Response_to_reviewers_PLOS_VF.pdf [file pgen.1011984.s015.pdf]

We thank the reviewers for their thorough evaluation of our manuscript and their detailed, helpful comments. Please find below a point-by-point response to all comments, including information about the changes we made in the manuscript.

Reviewer #1: The manuscript investigates the inter-individual variation in expression of *NRT2.1* in Arabidopsis thaliana individuals. LUC reporter activity is used as a proxy for *NRT2.1* expression and most experiments are done in seedlings grown on media. *NRT2.1* expression variability is investigated under different nitrate concentrations and followed intergenerationally and intragenerationally. *NRT2.1* expression is found to be variable between individuals and changes according to environmental conditions. This variability is further connected to three measured phenotypic traits. The claim of NRT2.1 expression variability is admitted to not be novel by the authors but the behaviors under changing conditions are.

The way the manuscript was written could have led to some confusion regarding the novelty of *NRT2.1* inter-individual transcriptional variability. No paper currently shows that *NRT2.1* has a high transcriptional variability. It is only by analysing the data available in Cortijo et al. 2019 that we determined this. We have now corrected the text in the manuscript to clarify this misunderstanding. (Lines 99 to 114)

If significant changes are made, including the inclusion of detailed study of similar genes, at least at the RNA-seq analysis level, this paper could be of broader interest to the field. My major reservations and comments are outlined below.
We thank the reviewer for all of their comments.

Line-by-line comments:
24 - "not transmitted to next generation" - please see my detailed comments below, this is not what was tested in the experimental setup.
We have changed every part of the manuscript related to this experiment so that it is no longer considered to be a study of a trans-generational effect. In the Abstract, we changed the sentence to say: "the expression level of *NRT2.1* in plants does not permit predicting its expression in the next generation". (Lines 25-26)

36 - "in this state" - Do not understand what state you are referring to
We changed the sentence to avoid the word "state", which is indeed vague and could create some confusion. The new sentence is: "Our study thus demonstrates that a global coordination of the genes involved in the carbon/nitrogen balance in plants is established in young seedlings, at different levels in each plant, and maintained over time." (Lines 30-33)

32-38 - The significance statement should be more specific; it could mention crops (like you do later) or at least the unanswered questions in the field you are trying to address
PLOS Genetics does not ask for a significance statement, which was a requirement for a previous submission in another journal. We want to apologise for keeping it in this submission. We have now removed it to follow the PLOS Genetics manuscript organisation, and have

added the Author summary instead, which also includes unanswered questions and puts our manuscript results in a broader context.

42 - "Phenotypes and gene expression" - Most consider gene expression to be a phenotype
We agree, and we have changed this to “morphological phenotypes and gene expression” in order to clarify what we meant. (Line 50)

42 - "Interestingly" - Please remove unnecessary linking words
47 - Remove "they"
We have revised the English of the entire manuscript to make it more readable. The new version of the manuscript was sent to a professional English editing service to improve its readability.

52 - What consequences? Positive and negative for what? Too vague.
We have made this clearer by enhancing the connection between: “The existence of this variability can have both positive and negative consequences, including for instance the suspected positive impact of seed germination variability on the survival of plant populations in the desert during unexpected droughts after a rainfall. On the other hand, variability in a range of phenotypes such as seed germination or flowering time is undesirable in crops, when trying to optimize farming procedures.” and the following sentences containing a list of positive and negative consequences of variability. (Lines 57-63)

58 - Expound on the mention of crops, it could be mentioned earlier, presumably it is the rationale behind your study
There might be a misunderstanding as the rationale behind this study is not to translate this work to crops, as there is still a lot to learn about variability. Rather, this is a basic science study of the regulation and role of inter-individual transcriptional variability in the model plant *Arabidopsis thaliana*, with limited reference to crops in the manuscript.

60 - Does selection happen at the level of the individual? Reference please. There is still significant debate in the field, please remove or justify your opinion.
Since there are articles that agree and disagree with this sentence, we have followed your first advice and simply removed it.

65 - "inter individual" - there is inconsistence in the spelling of inter-individual throughout the paper
We have corrected this throughout the manuscript.

83-98 - The justification for the use of NRT2.1 is a bit longwinded and does not explain why you did not select several genes in the same pathway or other highly variable genes.
We have now moved the justification of how we chose the gene used as a model in this study to the beginning of the Results section and substantially changed it. The criteria used to select a gene of interest are now detailed, explaining how *NRT2.1* was identified as a good candidate. (Lines 99 to 114)

101 - "as long as the gene is expressed" - nonsensical, rephrase or omit
We omitted this part of the sentence that was indeed confusing.

113 - "of" instead of "for"
The new version of the manuscript was sent to a professional English editing service to improve its readability.

115 - Please justify the use of media for most experiments (outside of nitrate concentration experiments), this seems like a major limitation. You did not compare the expression on soil and media despite transferring plants between the two in the "transgenerational" experiment.
We added a sentence to justify the use of plants growing in petri dishes and not in soil: "As *NRT2.1* is expressed in roots, all experiments were performed on plants grown in Petri dishes for easy imaging and access to the roots." (Lines 118-120)

117 - Please state the approximate number of seedlings per plate
We included the total number of plants used to measure the CV as well as the number of plants per plate: "Gene expression in individual seedlings was quantified during independent studies, each involving 24 to 32 seedlings (with 8 seedlings per plate)." (Lines 121-122)

140 - Figure S1A is your justification for the methodology used throughout the paper. It should be in the main text. Your 2 replicates have very different Rs - please address. I think at least one more rep is needed, and you need to address the variation in correlation per rep.
We have moved this to Figure 1 and included a third replicate. When re-examining the data, we realised one of the control genes used for the normalisation was contaminated. We only used the other gene to normalise expression, which solved the problem: all replicates now have a similar correlation.

162 - I am not convinced by the use of 0.5mM here when 1mM is used everywhere else. Is the "enhanced transcriptional response" not desired otherwise? Is there are reference for this?
We removed this experiment in order to streamline the manuscript and allocate space for other experiments and analyses requested by the reviewers.

168 - Please elaborate on this extrapolation. Is an uncoupling of the 2 the only possible explanation?
We have now changed the sentence to say: "This result shows that the *NRT2.1* expression level can change while maintaining its inter-individual variability." (Lines 157-159)

186-188 - "Loss of expression" - Are you talking about a complete loss of expression? If so, the loss of variability is agiven. If you are talking about very low expression levels, please clarify and propose hypotheses explaining the loss of variability at very low levels of expression.
We removed this experiment in order to streamline the manuscript and allocate space for other experiments and analyses requested by the reviewers.

194 - Fig. 2A - I am assuming there is no significant difference between the treatments. Please make this clear in the figure, the legend and in-text. It is an important caveat.
Reading your comment, we realize that we forgot to include the result of a statistical test showing that there is a difference in the *NRT2.1:LUC* signal between the two nitrate levels. This is now corrected in the figure. We also changed the text accordingly.

232-234 - What does this imply?
For clarification, we included the following sentence: "This indicates that the correlation between *pNRT2.1:LUC* and primary root length is a distinct property of the *NRT2.1* promoter, but not of the constitutive 35S promoter. This type of correlation must therefore be evaluated on a promoter to promoter basis, with *pNRT2.1* being a strong example of a positive correlation with primary root length." (Lines 203-207)

240 - Fig. 3 - The decision to group the data by replicates does not reveal the whole picture. What is the variance when you combine all 3 replicates? What is the variance when you compare the combined replicates between treatments? Is the difference in variance significant?
We decided to group the data by replicates to emphasise the fact that differences between the WT and *nrt2.1* mutant at 1mM KNO_3 were observed in all 3 replicates, despite differences in the absolute values in the three replicates. We now include a different statistical test to take into account possible differences between replicates, using Wilcoxon rank sum tests between all pairs of samples and adding the results of these tests as letters in the plot corresponding to the different identified groups.

288-300 - You attempt to investigate paternal effects, not transgenerational maintenance of expression levels. I have several issues with these experiments and would need to see it repeated or removed from the manuscript. The transfer to soil is a major conflating variable and you have no control for this and no separate experiment where you describe the assumed correlation between expression on media and soil. What if the act of transfer removes the variance even before selfing? Is there a reason you have separated the 2 experiments in Fig. 4D and S4F? Can you instead plot a scatterplot of all measurements as you have before, when you expected a correlation? You also don't discuss this potential "memory" or lack thereof in the discussion.
We have substantially edited that part of the Results, to remove all references to transgenerational maintenance of expression levels. We also took into account the fact that transferring plantlets to soil could affect *NRT2.1* expression, and accordingly adjusted the conclusion from this experiment. We also changed the figures to use scatterplots instead of boxplots.
Finally, we did specify that the two experiments were performed using different parents. This is why we kept the figures separate, as the parents for the two experiments were selected several months apart.

349-391 - You conducted whole genome RNA-seq and did extremely limited analysis on it. There are many more questions related to variability you could have addressed with this data. Please conduct a more detailed analysis.

We have now performed a more detailed analysis of the transcriptome to include:

- A thorough analysis of each cluster
- An analysis of the correlation between *NRT2.1* and cell wall-related genes, another GO enriched in the cluster containing *NRT2.1.* This analysis was complementary to the one on photosynthesis-related genes that was already present in the manuscript.
- A regulatory network centred around transcription factors, in order to find new regulators of *NRT2.1*

Consequently, we added a new figure and a new section in the Results.

We also want to specify that all transcriptomics studies were carried out to better understand NRT2.1 variability, which is why they are centred around that gene. In parallel, we are currently performing more general analyses of this RNA-seq, together with other datasets, which will be included in another manuscript. These include the analysis of TFs of interest derived from the network inference and the study of changes in variability levels in a mutant, which go beyond the scope of the present article.

402-403 - That is not what your experiment says.
We changed this sentence to say: "Furthermore, whereas the relative expression level of *NRT2.1* in seedlings is usually maintained for up to 3 weeks, it is not associated with the *NRT2.1* expression level in the next generation" (Lines 376-378)

416-418 - This is a major limitation and should be addressed earlier.
It is known that, despite this limitation, *NRT2.1* expression levels are correlated with the activity of NRT2.1 transporters, and we agree that our sentence did not make this clear. We have corrected it as follows: "While *NRT2.1* expression levels do not systematically correlate with the amount of NRT2.1 protein as it is subjected to post-translational regulation [47], it is known that *NRT2.1* expression levels are correlated with nitrate influx by high-affinity root nitrate mainly mediated by NRT2.1 [22]. We found that the expression of two genes coding for proteins involved in NRT2.1 post-translational modification (PP2C and NAR2.1) is positively correlated with *NRT2.1* expression in single seedlings" (Lines 402-407)

448-449 - Are you suggesting NRT2.1 expression variability is the only cause behind root length and density variability?
This is not what we meant to say. The text has been corrected to remove this misunderstanding.

Other general comments:
• To increase clarity and comprehension, I strongly suggest English language editing services. There are a lot of verbose sections with unnecessary linking words and some awkward grammar.
The new version of the manuscript has been revised by a professional English editing service.

• I think this single gene study could be very successfully expanded by including a few more genes (even without adding more phenotypic impact experiments). It would greatly expand the scope of the conclusions that can be drawn.

During the revision period, we analysed luciferase promoter reporter lines for other genes. However, as shown below, we found that their analysis did not merit including them in the manuscript. Indeed, identifying other genes to study, generating the luciferase promoter reporter lines and analysing them would require at least another year, which is not feasible for the revision of this manuscript.

The first reporter line we studied is *KTI1:LUC* (AT1G73260). Panel A in the figure below shows that the signal of this reporter line is very similar between plants, and that it substantially decreases after the heterotroph-to-autotroph transition (at around 4 days).
The second reporter line we studied is *ST4B:LUC* (AT1G13420). Although differences in expression can be seen between seedlings for this line, they are caused by the fact that we analysed the signal for the entire primary root, when that promoter is only active in the tips of primary and lateral roots. Signal peaks in the graph (panel B) correspond to the emergence of a new lateral root in that seedling. It shows the need of a temporal and spatial analysis of the expression of that gene, on top of the transcriptomic approach, in order to avoid misleading conclusions. This is also why we decided not to include any transcriptomic study centred around that gene in the revised version of the manuscript.

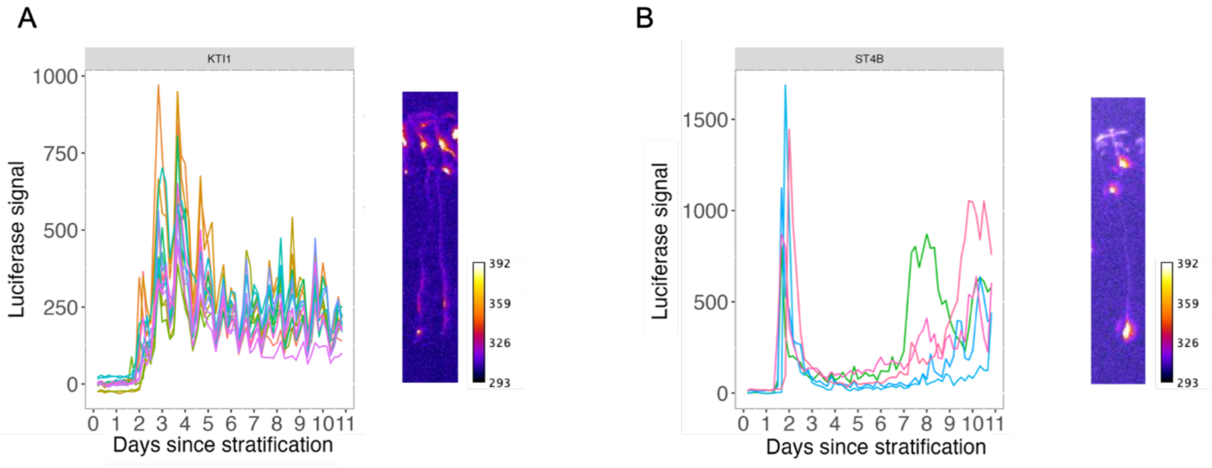

Figure legend: Luciferase signal over time for (A) KTI1 and (B) ST4B. Each line represents the signal in a given seedling, measured every 4 hours for 11 days after stratification. Images of signals for *pKTI1:LUC* and *pST4B:LUC* seedlings that completed the heterotroph-to-autotroph transition with overlaid bright field images (in grey scale) are also included. The colour scale of the luminescence signal, shown on the right of each panel, is the same in all images.

• There is no mention of circadian fluctuation in NRT2.1 expression, which is a major argument in the cited Cortijo et al. (2019). Was the imagining time controlled? Were steps taken to randomize the order of imaging of reps?
NRT2.1 expression is not regulated by the circadian cycle but depends on light. We have now included a sentence in the Material and Methods to indicate when plants are imaged: "Imaging was carried out around 8 hours after dawn, when expression for *NRT2.1* is high and stable, as its expression depends on the presence of light" (Lines 535-537)

• The Discussion is very verbose. The paragraphs are too long and poorly organized. Please

also discuss the potential epigenetic components contributing to expression variability in more details. A few sentences on the implications of the results would be great.
We have greatly reorganised the Discussion to improve comprehension, and we have included more details about potential epigenetic components as well as sentences about the implications of the Results.

Reviewer #2: The paper focused on one specific gene's inter-individual expression variability in the model plant *Arabidopsis thaliana* called NRT2.1. The central question of the paper is: when does inter-individual variability established and what are the consequences of this individuality? It's quite interesting to see that the individuality establishes at a young stage and maintain over days but not generations. However, the present form needs to be better organized and streamlined to increase the readability. In addition, some major results do not appear to have strong supports.

We thank the reviewer for their comments.
Following the suggestions of this and other reviewers, we have streamlined and reorganized the paper to make the story flow better.

Major comments:
The authors should leverage existing single-cell data to clarify the nature of NRT2.1 expression variability. Specifically, they could distinguish between two possibilities:
1. High cell-to-cell variability within each individual.
2. Relatively uniform expression among cells within an individual, but high variability between individuals.
If this is not feasible, could you discuss why? The relationship between cell-to-cell variability and inter-individual variability is a fun aspect to pursue.
We agree with the reviewer that this is a very interesting aspect to study, and one that we have explored. Using a reporter line that includes *pNRT2.1:mTurq* addressed to the nucleus as well as *p35S:H2B-RFP*, we analysed the cell-to-cell variability of these two reporters in the epidermis and the cortex, where *NRT2.1* is expressed. As shown in the figure below, the cell-to-cell variability of *NRT2.1* is comparable to *35S* variability within the cortex and atrichoblast cells. It is however higher in trichobast cells, where cells were observed with some *p35S:H2B-RFP* signal, but no *pNRT2.1:mTurq* signal.
While this result is interesting, we would need to perform the same experiment in several independent lines, and directly compare cell-to-cell and inter-individual variability in the same plants. The use of different genotypes that alter the level of *NRT2.1* inter-individual variability, or the study of many genes with high inter-individual variability, would be of great interest, but outside of the scope of this manuscript. Moreover, since the manuscript is focused on inter-individual variability, we think that adding this result might dilute our main message.

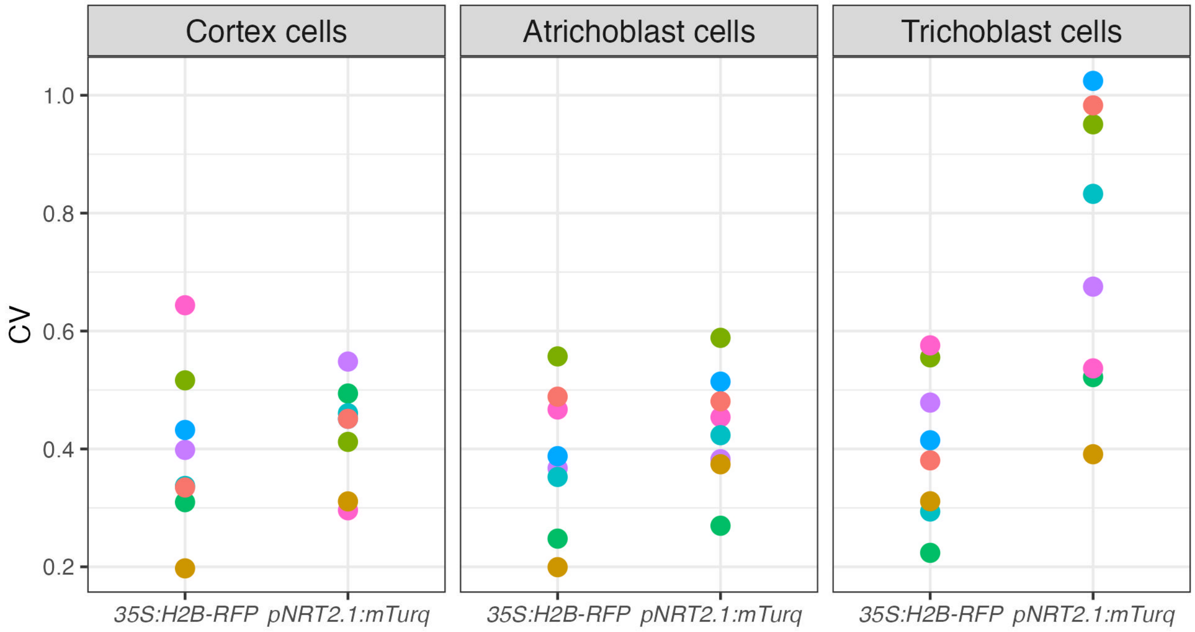

We would like to add that analysing cell-to-cell variability using publicly available single cell RNA-seq datasets is currently not possible, as this is based on pooling multiple plants. With these datasets, we cannot discriminate between cell-to-cell variability and inter-individual variability. In order to do so, we would need to perform scRNA-seq on single plants, which has not been done yet to our knowledge.

It appears that the gene with higher variability also exhibit high variation of (unstable)CV between experiment, making it difficult to get conclusion with CV. What's the potential cause of this? Does this suggest the sample size 24 seedlings is insufficient to capture CV. Similarly for Figure S1C, Within each environment, is the variation of CV among 23 experiments high? Any hypothesis of why and potential implications for the entire manuscript?
This is a very valid point, as we currently do not know how many plants are required in each independent assay to obtain a reproducible measure of variability. A sample size of 24 to 32 plants per assay is a compromise to ensure that all plants are measured within a reasonable time frame (2 hours maximum when several environmental conditions are analysed), but it might still not be enough. To highlight this limitation, we performed a higher number of independent assays than the research community standard, in Figures 1 and S1. In addition, we now include a paragraph in the Discussion to take this point into consideration (Lines 386-396). Nevertheless, all assays in Figure S1B were performed over a period of 3 years by four different people on different seed batches and with two bioluminescence cameras. Consequently, we cannot exclude that part of the variation in CV between assays could result from this.

Thus, I question whether the results from Fig1D and 1F are robust - it seems apparent from 1A that after the decrease of CV upon starvation, the CV is still within the range under normal conditions. I suggest that the authors enhance the robustness of the results and move this section to Supplemental materials. or alternatively, remove this section if the results do not have strong support.

We removed the experiments corresponding to Fig1d-D and 1F (response to changes in KNO_3 concentrations) in order to streamline the manuscript and include additional experiments and analyses.

It seems the experiments of figure 1 and 4 are similar. Why don't you use the data from figure 1 (transfer media) to answer the question regarding the effect of environmental fluctuation in relative expression level? I suspect that the samplings of temporal intervals maybe different? Thus, it would be better to have a clear cartoon diagram for figure 1 and 4 to show the design of experiment and clarify on the number/label of plate and number of seedlings/individuals. I'm not sure I understand why Fig4C represents 19 seedlings?
We have reorganized the order of figures and removed the experiments corresponding to Fig1d-D and 1F, which solved this problem. Also, there was a mistake in the legend of Fig 4C, which has been corrected.

Minor comments:

For Fig 1A, what's the specific gene for HVG and LVG?
We now include more information about these genes in the manuscript: "For comparison, we also selected two control genes (AT1G08930 and AT2G28810) from the previously mentioned study on inter-individual variability for the entire transcriptome of *Arabidopsis thaliana* [9] and in which their level of variability was validated by RT-qPCR. Both genes are expressed throughout the entire day/night cycle, and AT1G08930 is a highly variable gene (HVG) during the day, whereas AT2G28810 is a low-variability gene (LVG) at all time points." (Lines 109-114)

Line 191 The section titles is a little bit vague in a sense it could also just imply a genotype with higher(lower) expression variability exhibit higher(lower) phenotypic variability without correlated expression and phenotypes. Perhaps replace/remove 'variability'
We have changed the title of this section to: "NRT2.1 expression differences between plants correlate with different phenotypes". (Line 161)

Line 288 relative NRT2.1 expression level?
This has been corrected.

Line 467 - 472: the statement read as if they are contradicted. Perhaps increase → decrease?
Thank you for this suggestion, however this experiment has been removed.

FigS1C. What is a replicate? a relevant question is what do you mean by 'combining 23 experiments' in Line 154. What are differences between experiments? Could authors clarify a bit in the results section?
We have clarified what replicates are throughout the manuscript by adding the following sentence in the Material and Methods: "Replicates for all experiments consisted in fully independent experiments performed on different dates with independently grown plants." (Lines 526-527)

Fig 4D caption. the parents selected in (B) → selected in (C)?

Line 321 NRT21 → NRT2.1
Line 360 **$R^2 > 0.7$?**
These 3 mistakes have been corrected.

Reviewer #3: Lecuyer, Vettor et al. is an interesting study in which the authors have gone in depth to analyze inter-individual variability in the expression of NRT2.1, a nitrate transporter gene, in Arabidopsis seedlings. The analysis is quite exhaustive following a sequential series of experiments which reveal unexpected and interesting findings about the nature of inter-individual variability, such as the establishment of variability in early seedlings but not transmitted to the next generation. Experiments of phenotypic consequences mediated by NRT2.1 provide a great complement to the initial findings. The authors analyse correlation between NRT2.1 expression levels and genes involved in nitrate metabolism and then expand the correlation analysis to genome-wide data showing an enrichment of correlated genes involved in photosynthesis.

I find the study attractive and appealing, and I find it will be of interest to different sectors of the community. The experimental findings are robust and I find the video provided a great example of how we need to adopt new forms of sharing results. The network findings are somewhat limited and I invite the authors to go a bit further in that direction to strengthen the message of their manuscript. I also point out some major and minor issues that I consider should be addressed.

We thank reviewer #3 for their comments.

Major comments:
I find that the last sentence of the abstract does not reflect the findings of the manuscript accurately. Please consider rephrasing it.
We have changed the end of the Abstract to reflect the changes made in the manuscript, and now include the sentence: "This work shows that thanks to single-plant analysis of gene expression, we can gain new knowledge on the mechanisms behind a phenotype of interest that is normally masked in studies performed on pooled plants." (Lines 34-37)

Section: New factors involved in regulating NRT2.1 expression level in seedlings
I am not convinced with the conclusions of the last results section and I believe the data can be exploited a bit more. In particular, I am not convinced that taking the percentage of genes from each pathway with a correlation with NRT2.1 expression with a p-value below 0.05 is an appropriate measure. Arguably, there seem to be many points close to the 0.05 threshold for photosynthesis related genes. The authors could consider using a volcano plot-like figure (correlation value vs. p-value) and take the percentages from there. That said, the size and diversity of the pathways might also influence this result so the percentage might not be accurate even in this case. Additionally, even if the percentage of genes involved in nitrogen nutrition is smaller, their correlation values seem to be higher.
Thank you for this suggestion. We now represent the data as a volcano plot as proposed, which makes the results clearer without drastically changing the conclusion. We also highlighted in the figure the genes from nitrogen nutrition that have a high correlation, and showed that none of them are regulators of *NRT2.1* expression.

Are the genes with high correlation found in cluster 1? If they are not in cluster 1, it would imply that the results from the clustering (6A) are not revealing genes that are correlated, which would be a problem. It could be that cluster 1 is grouping highly variable genes regardless of their correlation, for example, but this does not seem to be the case according to Figure S5C, so I feel I am missing something here. If they are in cluster 1, it would imply that the results from GO enrichment (6B) are not that relevant to reveal underlying regulatory pathways. Additionally, what is the overlap between the list of nitrogen nutrition genes used in figure 7C and Figure 6? I would invite the authors to explore this a bit further to strengthen the conclusions.
We now include a volcano plot to analyse the correlation of genes in each cluster with *NRT2.1*. As seen in Figure S7, the majority of the genes in cluster 1 have a statistically significant positive correlation with *NRT2.1*. However, most of the genes in the different pathways we analysed are not in the hierarchical clustering, as their CV^2 is lower than 1. Indeed, in order to maximise our chance of finding patterns, we only kept genes with $CV^2>1$, representing around 1700 genes (approximately 10% of the studied transcriptome). This allowed us to run the hierarchical clustering and identify GO of interest. We then took all genes for each pathway, regardless of their CV, and analysed their correlation with *NRT2.1*.

I find that the findings of early establishment of NRT2.1 expression variability, reversal after change in conditions but no trans-generational inheritance is very interesting and are essential results of the paper, in my opinion. I was somewhat expecting a bit more discussion in this regard and would invite the authors to do so. I consider the discussion of the paper would benefit greatly.
Since reviewer #1 pointed out that the experiment is not trans-generational, which we agree with, we did not include more on this subject in the Discussion. However, we added other points to the Discussion from the more detailed analysis we made of the RNA-seq data.

I think the paper would benefit from an effort to homogenize the figures. I feel there is an excess of diversity in terms of font sizes, color schemes, header colors, etc. This could be subjective, of course, but my comment is intended to make the figures clearer. Lack of consistency exists even within figures, which is unnecessary and does not help in transmitting the information adequately. As an example to make a point, the differences between axis labels, axis label fonts, and headers between figure 5A and figure 5B are striking, when they are supposed to be the same type of figure and show the same type of data. Differences between Figure 5A and Figure S3A are also unnecessary. There is a mistake in the naming of supplementary figures (e.g. Figure S6 is in reality Figure S5).
We homogenised all figures in term of colours and font size, and checked all figure references in the text and legends.

You might want to reconsider your main vs supplementary figure choice. For example, consider merging current Figures 2 and 3 and including current Figure S5C in Figure 7.
We have taken this suggestion into account and greatly reorganised the figures to include the graphs showing the correlation with *NRT2.1* in cluster 1 as part of a main figure.

Data availability: Although the authors might have strong reasons not to release the raw data generated for this study, I do not see any strong reasons not to make their code available to

be reviewed. Given the importance of bioinformatic code for some of the results of this manuscript I would strongly encourage the authors to make their data available prior to acceptance or point to strong reasons why not to. That said, if there are no strong reasons to withhold the raw data prior to acceptance, please reconsider doing so.

Data were deposited at data.recherche.gouv.fr, with access granted to reviewers thanks to this confidential URL:
https://entrepot.recherche.data.gouv.fr/privateurl.xhtml?token=993aaf7b-50f5-47ad-911d-21824a3f0ca7

Minor comments:

What is the rationale behind the choice of AT1G08930 as HVG and AT2G28810 as LVG? Was RT-qPCR performed de novo for these genes for this work or are the data from Cortijo et al. 2019? Please specify. Were these three the only genes analyzed? If this is de novo work, how do CV values compare to those from Cortijo et al. 2019. I ask this because it would provide more strength to the choice of these genes. With the current writing, it seems arbitrary when I imagine it is not.

We have now included more information about these genes in the manuscript: "For comparison, we also selected two control genes (AT1G08930 and AT2G28810) from the previously mentioned study on inter-individual variability for the entire transcriptome of *Arabidopsis thaliana* and in which their level of variability was validated by RT-qPCR. Both genes are expressed throughout the entire day/night cycle, and AT1G08930 is a highly variable gene (HVG) during the day, whereas AT2G28810 is a low-variability gene (LVG) at all time points." (Lines 109-114)

Although I appreciate the concluding sentence in each section, I feel that "all in all" is not the most appropriate. Given that the findings are sequential, it might be nice indeed to have a conclusion in each section. I would suggest something like "Finding:" or "Conclusion:" prior to each concluding sentence that stands out. In the current form, the fact that there are short one or two sentence paragraphs at the end of each section is a middle ground that I don't find convincing.

We decided to remove the conclusion sentences at the end of each section.

Figure 2: I suggest using the same color scheme for B as in C and D. "Primary". Clarify units for lateral root length.

We used different colours as different replicates are used in Figure B compared to Figures C and D. We added how the lateral root density is calculated.

Noting that NRT2.1's ID is AT1G08090 would be nice in the main text. Additionally, the expression pattern of NRT2.1 across organs according to TAIR seems to be specific to seedlings and roots. It would be nice to point that out.

We have added that *NRT2.1* is AT1G08090, and that this gene is expressed only in roots. However, data from several papers (Okamoto et al., 2003; Cerezo et al., 2001; Jacquot et al., 2020) show that it is not specific to seedlings, but also expressed in older plants.

Line 344: Please list the genes analyzed.

The list of analysed genes, and whether they are detected as an HVG or not, are now included in a supplementary table. We also included two other supplementary tables containing the list of genes in the different clusters and modules respectively.

Figure 6: A bit of more work on this figure would make it much more appealing. There seems to be a detachment between the text and figure 6A. Please make the figure legend fit the in-text description. Please clarify that CV2 is used in this case instead of CV (also in the figure legend). Figures 6B-D: consider adding correlation values or visualize it in a way that this is possible. Using a color code that merges Figure 6A with figures 6B-D would be helpful. For example, it is not clear to me why there are only 2 orange points in Figure 6A, but 4 genes in Figure 6B.
We have corrected text and figures to specify that CV^2 is used, thank you for pointing that out. We also used matching colour codes between Figures A and B. Genes that are detected as HVG are indicated in Figure A. Also, genes that significantly correlate with *NRT2.1* are now shown in bold in the figure. In Figure A (CV^2 VS mean expression), very lowly expressed genes were removed as they pose a problem for detecting HVG. However, they are present in the correlation analysis in Figure B. This explains why some genes are missing in Figure A as compared to Figure B.

Figure 7B: I also note some detachment between what I see in Figure 7B and what I read in the text. For example, plant-type cell wall organization is not mentioned when it appears to have the highest -log10(FDR) value. The following work, measuring inter-individual variability in Arabidopsis sepals, might be relevant in this regard: Hartasánchez et al. 2023: https://doi.org/10.24072/pcjournal.327.
We have now included an analysis of cell wall-related genes and included a paragraph in the Discussion about them, citing the Hartasánchez reference.

Lines 433-458: I find this paragraph hard to follow. It seems to be going back and forth and the main message is lost. Consider rephrasing the whole paragraph.
This paragraph has been substantially rewritten. (Lines 420-432)

The manuscript would benefit from a stringent grammatical review including figure legends. I point out some of the issues I found below. I suppose there are more that I did not see.
These have all been corrected. Moreover, the manuscript has been proofread by an English editing service.
Line 74: “ones”
Line 85: “First of all,” and provide citation for previous study
Line 107: “photosynthesis,”
Line 115: “grown on media”
Line 117: “the plant’s”
Line 118: “(CV), that is,”
Line 137: “could” instead of “can”
Line 138: “the NRT2.1”
Line 145: “described above”
Line 146: “However,”
Line 153: Not clear what “as already published” means here

Line 154: “seedlings” and “seedlings”
Line 160: “to” instead of “in”
Line 162: “concentration, with”
Line 163: “respectively, for this experiment specifically” and “saw”
Lines 164 and 165: “a medium” or “media” without the “a”
Line 166: specify that this is CV, just for completeness
Lines 217, 353, 360: “Spearman”
Line 269: “had” instead of “have”. In general, please check for consistency of verbal tense across the manuscript (e.g. line 315, 325)
Line 288. “Is” instead of “are”. In general, please check for consistency in singular versus plural. Consider using “expression levels in seedlings” instead of “level” but try to be consistent.
Line 309: check parenthesis
Line 318: check full stop
Line 362: “known that the”
Line 400: “influx of nitrate by high a affinity transporter system as well as primary root length and lateral root density”
Line 434: “primary root length and lateral root density”
Line 437: “GO terms associated with” instead of “GO involved in”
Line 441 and 446: “low nitrate conditions” or “a low nitrate condition
Line 444: “these” instead of “the 2”
Line 477: “did not”
Line 485: provide citation here
Line 535: “Petri”
Line 620: “Author contributions”
