# Supplementary material for: Establishment and maintenance of NRT2.1 inter-individual variability in plants
Source: PLoS Genet. 2025 Dec 17;21(12):e1011984. doi: 10.1371/journal.pgen.1011984 (PMC12742734; doi:10.1371/journal.pgen.1011984)
Supplement: Attachment — Submitted filename: Response2_to_reviewers_PLOS.docx [file pgen.1011984.s016.docx]

We thank the reviewers for their thorough evaluation of our manuscript and their helpful comments. Please find below a point-by-point response to all comments, including information about the changes we made in the manuscript.

Reviewer's Responses to Questions

**Comments to the Authors:
Please note here if the review is uploaded as an attachment.**

Reviewer #1: The authors have substantially improved the manuscript and adequately addressed all of my concerns.

We thank the reviewer for their comment.

Reviewer #2: The revised manuscript by Lecuyer et al. represents a substantial improvement over the initial submission, with significant text revisions and reorganizing figures. The revised version now provides a more cohesive narrative on how NRT2.1 variability arises and maintained within, but not across, generations.

We thank the reviewer for their comment.

I would add that regarding the discussion around the regulator of NRT2.1, the determining factor of expression variability does not have to come directly from upstream transcriptional factors. For example, the cis-regulatory region (some cis motif, such as TATA box, are inherently sensitive to subtle environmental changes and hence noisy), epigenetic factors etc. Some epigenetic factors can regulate expression level within generations but not across as well.

You are right, we have included the following sentence in our discussion to also consider the role of other factors in *NRT2.1* inter-individual transcriptional variability: “Our results could also suggest that *NRT2.1* high inter-individual transcriptional variability is influenced by other genomic and epigenomic factors such as the TATA box or chromatin modifications, which were shown to regulate variability in expression in other organisms.” (lines 476-479)

Reviewer #3: The authors have performed a substantial revision of their manuscript, increasing clarity in the main message, addressing limitations of their research, and providing more detail in areas where it was lacking. They have addressed all of my comments and suggestions as well as those of the other reviewers. The manuscript is also much better in terms or readability and in consistency across figures and text. I particularly appreciate the modifications and connection between current Figures 5 and 6. I just have a comment on Figure 7 and a few additional minor comments regarding small details on the text.

We are grateful for the reviewer’s feedback

Main comment:
I recognize that the network analysis might have been done following a suggestion on my part and I appreciate it, yet, with the addition of Figure 7 and the network analysis, new questions come to mind. If I understand correctly, the same genes for the hierarchical clustering are used for the DIANE network. I also understand that DIANE focuses on transcription factors and the finding of PIF8 is relevant. However, Figure 7B shows correlation between NRT2.1 and genes in module 6. I understand that these reveal co-expression. So, my question would be, what is the overlap between cluster 1 and module 6? Why are the GO enrichment results so different? It could be that module 6 is smaller than cluster 1 and hence has lower statistical power, but in any case, this discussion is currently missing from the manuscript and seems to be important.
At this moment I am not sure what I would recommend. One possibility could be to reduce the DIANE results to a mention of PIF8, discussing the other modules less and reducing this bivalence between hierarchical clustering clusters and network modules. Another possibility could be to compare networks and modules, or at least give reasons as to why the results differ. Additionally, for a reader not familiar with DIANE, it is not clear what is meant in lines 365-368 with “UNE10 is the only TF connected to NRT2.1”. Please be more specific. In that regard as well, a section on network reconstruction with DIANE is missing from the material and methods section. To be clear, I think this addition is nice but the way it is presented now adds more noise than clarity. I think the manuscript would benefit from either reducing or increasing this particular section, although the PIF8 finding and discussion should definitely be kept.

Thank you for your suggestion on how to improve this new section of the results. We decided to reduce the importance of the network inference and only focus on the discovery of a possible new transcriptional regulator of *NRT2.1.*

We included the following sentence to explain what we mean by “UNE10 is the only TF connected to NRT2.1”: “This means *PIF8* is the only transcription factor with a statistically significant correlation with *NRT2.1* to be included in the regulatory network. This indicates that *PIF8* might regulate *NRT2.1* expression, so that it would need to be tested experimentally.” (lines 361-363). We also included the network inference in the material and methods.

Other minor comments:

Thank you, we have changed the text according to all of the following comments
Line 213. Define WT as wild type.
Line 333. Gene expression “levels”
Lines 343 & 556. Possible double space?
Line 367. No study “has” explored
Line 394. And “of” the minimum number of plants. - to facilitate reading this sentence
Line 411. NRT2.1 “expression”
Line 411. I find that “when it comes to the control of their expression” is not needed, although I might be missing something here
Line 422. Define KO or “knockout”

Line 426. “a growth medium” or “growth media”

Line 426. Define C/N

Line 466. Cite literature for known transcriptional regulators again (34-37 ?)

Line 475. “or DAP-seq”

Line 591. Capital letter “Pearson”

Line 589. “Z-score” (or Zscore if you prefer, but be consistent across the text and figure legends- 5B and 6 for example)

Line 590. Please correct the expression by adding parenthesis: ((CPM of a gene - mean expression of that gene in samples)/standard deviation of the expression for that gene in samples)

Line 955. “as squares”
Lines 973 and 979. GO “terms”
Figure 6D. “GO enrichment”
